# First record of *Aspergillus nomiae* as a broad-spectrum entomopathogenic fungus that provides resistance against phytopathogens and insect pests by colonization of plants

**DOI:** 10.3389/fmicb.2023.1284276

**Published:** 2024-01-08

**Authors:** Zhengkun Zhang, Yifan Tian, Li Sui, Yang Lu, Ke Cheng, Yu Zhao, Qiyun Li, Wangpeng Shi

**Affiliations:** ^1^Institute of Plant Protection, Jilin Academy of Agricultural Sciences, Changchun, China; ^2^Jilin Key Laboratory of Agricultural Microbiology, Changchun, China; ^3^Key Laboratory of Integrated Pest Management on Crops in Northeast China, Ministry of Agriculture and Rural Areas, Changchun, China; ^4^Department of Entomology, College of Plant Protection, China Agricultural University, Beijing, China; ^5^Jilin Agricultural Science and Technology University, Jilin, China

**Keywords:** entomophagous fungus, *Aspergillus nomiae*, endogenous colonization, plant disease resistance, biological control

## Abstract

**Introduction:**

*Aspergillus nomiae* is known as a pathogenic fungus that infects humans and plants but has never been reported as an entomophagous fungus (EPF) that can provide other functions as an endotype.

**Methods:**

A strain of EPF was isolated and identified from diseased larvae of *Spodoptera litura* in a soybean field and designated AnS1Gzl-1. Pathogenicity of the strain toward various insect pests was evaluated, especially the ability to colonize plants and induce resistance against phytopathogens and insect pests.

**Results:**

The isolated EPF strain AnS1Gzl-1 was identified as *A. nomiae*; it showed strong pathogenicity toward five insect pests belonging to Lepidoptera and Hemiptera. Furthermore, the strain inhibited the growth of *Sclerotinia sclerotiorum in vitro*, a causal agent of soil-borne plant disease. It colonized plants as an endophyte via root irrigation with a high colonization rate of 90%, thereby inducing plant resistance against phytopathogen infection, and disrupting the feeding selectivity of *S. litura* larvae.

**Discussion:**

This is the first record of a natural infection of *A. nomiae* on insects. *A. nomiae* has the potential to be used as a dual biocontrol EPF because of its ability to not only kill a broad spectrum of insect pests directly but also induce resistance against phytopathogens via plant colonization.

## 1 Introduction

The long-term and large-scale use of chemical insecticides not only enhances the physiological resistance of insect pests but also affects the population of important natural enemy species that cause environmental pollution, imbalance of the field community structure, and other negative effects on the ecosystem (Karthi et al., 2018). In the theory of integrated pest management (IPM), entomophagous fungi (EPF) have become one of the most critical tools in the population control of agricultural insect pests and are considered safe alternatives to chemical insecticides (Masoudi et al., 2018), which can directly infect and penetrate the host and cause disease (Arunthirumeni et al., 2023). It is more interesting that EPF can control insect pests by a sustainable effect, due to which they can continue to grow until the hyphae invade all of the tissues and organs and penetrate the host cuticle to produce conidia. After the death of the insect, the conidia can form a new infection cycle by natural transmission and then infect other insect hosts (Conceschi Marcos et al., 2016; Mora Margy et al., 2018).

Currently, more than 1,000 species of EPF have been reported, including commercially applied *Metarhizium anisopliae*, *Beauveria bassiana*, *Isaria fumosorosea,* and *Metarhizium rileyi* (Kim Jae et al., 2013; Grijalba Erika et al., 2018; Jiang et al., 2019; Malinga Lawrence and Mark, 2021; Wang et al., 2021; Acharya et al., 2022; Faria et al., 2022; Im et al., 2022), as well as literature reported *Paecilomyces* var*iotii*, *Hirsutella citriformis*, and *Akanthomyces lecanii* (Gandarilla-Pacheco et al., 2013; Cortez-Madrigal et al., 2014; Hussain et al., 2018; Naeem et al., 2020). However, the resources of EPF are insufficient due to their host specificity, which could affect their commercial production and application (Wichadakul et al., 2015; Zhang et al., 2020; Islam et al., 2021). Therefore, it is of significance to obtain new EPF strains with high pathogenicity against insect pests for the development of fungal insecticides.

In recent years, EPF strains with multiple control effects on plant disease and insect pests are gradually becoming a research hotspot, which means that the EPF can not only directly kill insects, but also form symbioses with plants through endophytic colonization, via various methods including foliar spraying, seed treatment, root irrigation, and injection (Ownley et al., 2008; Greenfield et al., 2016; Bamisile et al., 2019; Hu and Bidochka, 2021). It is reported that some EPF strains can promote the biomass of plants after colonization (Bamisile et al., 2018; Deb et al., 2022; Altaf et al., 2023), especially having the ability to affect the growth and development of pests and subsequently reducing their survival and reproduction (González-Mas et al., 2021; Altaf et al., 2023). Moreover, the colonization of EPF in plants can inhibit phytopathogen growth and induce plant resistance against infection (Barra-Bucarei et al., 2019; Gange et al., 2019; Canassa et al., 2020).

In the present study, a strain of EPF was isolated from muscardine cadaver larvae of *Spodoptera litura*, which was collected from a soybean field in Jilin Province, China, and characterized by morphological and molecular methods. Pathogenicity of the EPF isolates toward important Lepidoptera larvae, including *S. litura*, *Chilo suppressalis*, and *Ostrinia furnacalis,* was tested, along with Hemiptera pest adults, *Rhopalosiphum padi* and *Acyrthosiphon pisum*. The endophytic colonization ability in soybean plants, the feeding selection effect on *S. litura* larvae, and the inhibitory effect on the phytopathogenic fungus *Sclerotinia sclerotiorum* induced by colonization were explored. The findings provide new resources for the development of biopesticides with multiple control measures against plant disease and insect pests.

## 2 Materials and methods

### 2.1 Insects, plants, and phytopathogen strain preparation

Muscardine cadaver larvae of *S. litura* were collected from a soybean field in Gongzhuling City, Jilin Province, China (N43° 50′42″, E124° 82′58″).

Tested insects including Lepidoptera pest larvae *S. litura*, *Chilo suppressalis,* and *O. furnacalis*, as well as Hemiptera pest adults *R. padi* and *A. pisum*, which were maintained on regular artificial diets, were provided by Professor Wenjing Xu from the Plant Protection Institute of Jilin Academy of Agricultural Sciences.

Commercialized Jiyu 303 soybean seeds were bred by the Soybean Research Institute of Jilin Academy of Agricultural Sciences.

The *S. sclerotiorum* strain isolated and characterized from soybean was provided by Professor Jinliang Liu, Jilin University, China.

### 2.2 Isolation and culture of the EPF strain

Muscardine cadaver larvae of *S. litura* were disinfected with 75% (v/v) anhydrous ethanol for 30 s and with 1% (v/v) sodium hypochlorite for 2 min; rinsed with sterile water three times; the surface water was absorbed with a sterile filter paper; cut into five sections using a sterile scalpel; placed on Potato Dextrose Agar (PDA) medium (Sangon Biotech, Shanghai, China), which contained 1 mL ampicillin per liter at a concentration of 100 μg/mL; and incubated in a constant temperature incubator at 26°C for 5–7 days until mycelium grew. The strain was purified by the single-spore isolation method as described previously (Ghalehgolabbehbahani et al., 2022), and the purified strain was designated AnS1Gz1-1.

### 2.3 Morphological identification of the EPF strain

Monospores of the purified strain were picked and inoculated on PDA medium, and plates were incubated in a constant temperature incubator at 26°C. When spore mounds were observed with the naked eye, the morphology of conidia and hyphae was observed and recorded under a light microscope LW300-48LT (Siwei Optoelectronic Technology Co., Ltd., Xi’an, China).

### 2.4 Molecular identification of the EPF strain

Purified monospores were inoculated on the PDA medium for 7 days until sporulation, conidia were collected and suspended in a sterile 0.1% (v/v) Tween-80 solution at a concentration of 1 × 10^8^ conidia /mL, evenly applied to the culture medium, and incubated in a constant temperature incubator at 26°C for 10 days. Fungal bodies were scraped off using a sterile glass slide for DNA extraction according to the description provided by Watanabe et al. (2010). Three genes were employed for molecular identification of the EPF strain according to Hong et al. (2005) by PCR amplification, including the internal transcribed spacer (*ITS*) gene with primers ITS1 (5′-TCCGTAGGTGAACCTGCG-3′) and ITS4 (5′-TCCTCCGCTTATTGATATGC-3′), a segment of the β-tubulin gene (*BenA*) with primers βtub1(5′ -AATTGGTGCCGCTTTCTGG-3′) and βtub2 (5′-AGTTGTCGGGACGGAATAG-3′) (Balajee et al., 2005), as well as a segment of the calmodulin gene (*CaM*) with primers cmd5 (5′-CCGAGTACAAGGAGGCCTTC-3′) and cmd6 (5′-CCGATAGAGGTCATAACGTGG-3′) (submitted separately to GenBank with the accession numbers OR801769, OR801770, and OR801771). The PCR reaction systems (30 μL) included 15 μL of PCR Mix (Nanjing Novozan Biotechnology Co., Ltd.), 11 μL ddH_2_O, 1.5 μL forward primer, 1.5 μL reverse primer, and 1 μL genomic DNA. Thermal cycling was performed as follows: initial denaturation of 94°C for 3 min, 30 cycles of 94°C for 1 min; 55°C for 30 s, and 72°C for 30 s, respectively; followed by a final extension period of 72°C for 5 min. The PCR products were cloned in a pMD18-T vector (Takara, Dalian, China) and transferred into *Escherichia coli* DH5ɑ. The plasmid DNA isolated from Luria-Bertani cultures was sequenced in both forward and reverse directions by Sangong Biotech Ltd. (Shanghai, China). The forward and reverse sequencing results were spliced using DANman software (V6.0.3.99). The sequences of the three genes were submitted to GenBank separately to obtain the accession number. The phylogenetic trees were inferred by using the maximum likelihood method with a bootstrap consensus tree inferred from 1,000 replicates (Thompson et al., 2003). The evolutionary distances were computed using the Kimura 2-parameter method. The phylogenetic tree was conducted in MEGA X software (MEGA Inc., Englewood, United States). The tree was rooted to *Talaromyces flavus* CBS 310.38.

### 2.5 Evaluation of virulence against insect pests

Cultivated conidia on PDA medium were suspended in sterile 0.1% (v/v) Tween-80 solution at a concentration of 1 × 10^8^ conidia /mL, and sterile 0.1% (v/v) Tween-80 served as a control. Uniformly sized second instar larvae of *S. litura*, *C. suppressalis,* and *O. furnacalis*, as well as adults of *R. padi* and *A. pisum,* were selected for virulence determination by impregnation assay as described previously (Faria et al., 2022). After inoculation, larvae of *S. litura*, *C. suppressalis,* and *O. furnacalis* were placed in a 24-well culture plate, while *R. padi* and *A. pisum* were placed in sterile culture boxes and maintained at 25 ± 1°C with 70 ± 1% relative humidity and a 16 h light:8 h dark photoperiod. Three repetitions were performed with each treatment including 20 insects. Infection and death of insects were assessed every 24 h, as were the external characteristics of infected insects. Insects were considered dead if there was no sign of activity after gently touching the inoculated insect body with sterile forceps. Corrected mortality rates for each treatment group were recorded, and probit linear regression analysis was employed to calculate the median lethal time (LT_50_) of tested fungi against different insects as described previously (Liu et al., 2022) using the following formula:

Corrected mortality (%) = (mortality of treatment − mortality of control)/(1 − mortality of control) × 100.

Dead insect bodies were removed in a timely manner using sterile tweezers and placed in a sterile culture dish (ɸ = 9 cm) lined with sterile filter paper. Insect bodies were cultivated in a light incubator at 25 ± 1°C with 70 ± 1% relative humidity and a 16 h light: 8 h dark photoperiod. Growth of mycelium and conidia on the surface of insect bodies was observed, and the morphology of mycelium and conidia was examined under a microscope (Leica TCS SP8, Leica Microsystems, Wetzlar, Germany).

### 2.6 Evaluation of *in vitro* antagonistic activity of the EPF strain

The plate confrontation method was employed to assess the *in vitro* inhibition activity of tested fungi against *S. sclerotiorum* as described previously (Nzabanita et al., 2022). A 5-mm-diameter fungal block was taken from the edge of the active growing fungal pathogen colony (cultured for 5–7 days on PDA medium) and inoculated in the center of a PDA plate. Two 5-mm-diameter fungal blocks from the AnS1Gzl-1 strain cultured for 5–7 days were inoculated at a distance of 1.5 cm on both sides of the fungal pathogen block on the same line, while the control group was only inoculated with the fungal pathogen block, the plates were cultured at 26°C, observed and photographed at 5 days post-inoculation. Each treatment and control were repeated five times.

### 2.7 Evaluation of *in vitro* pathogenicity of AnS1Gzl-1 strain to soybean

Twenty surface-sterilized soybean leaves were selected, 10 leaves were used to inoculate with AnS1Gz1-1 strain, and the others were used as control. The selected soybean leaves were surface-sterilized as described previously (Sui et al., 2020). The trifoliate leaves were removed with sterile scissors and placed on a culture dish with moist filter paper. The moist cotton ball was placed at the petiole for moisture, and 5-mm-diameter fungal blocks of AnS1Gzl-1 strain were used for leaf surface inoculation (avoiding leaf veins), which were collected from the edge of the active growing fungal pathogen colony cultured for 5–7 days on PDA medium. The remaining 10 leaves were inoculated with blocks of culture medium without pathogen inoculation as a control. The fungus-inoculated and medium-inoculated leaves were incubated at 25 ± 1°C with relative humidity maintained above 70% and a 16 h light:8 h dark photoperiod. The symptom of soybean leaves was observed and recorded for 3 days after inoculation.

### 2.8 Colonization of the EPF strain in soybean plants via root irrigation

Healthy soybean plants with consistent growth at the compound leaf stage were selected and irrigated with a conidia suspension (1 × 10^8^ conidia/mL) of strain AnS1Gzl-1. A 0.1 (v/v) Tween-80 solution served as a control. Each treatment included three replicates with 20 seedlings per replicate, and the volume of the root irrigation solution was 20 mL. The colonization rate of strain AnS1Gzl-1 in soybean leaves was assessed by observation of fungal colonies grown from plating surface-sterilized leaf segments on PDA for 14 days as described previously (Sui et al., 2020).

### 2.9 *In vitro* evaluation of plant disease resistance induced by fungal colonization

For *in vitro* disease resistance evaluation, there were four treatment groups. The soybean seedlings were treated by conidia suspension (1 × 10^8^ conidia/mL) of AnS1Gzl-1 strain and 0.1 (v/v) Tween-80 solution via root irrigation, and the colonization seedlings were verified according to the above description. The second healthy soybean trifoliate leaves from top to bottom of seedlings that were positively colonized by AnS1Gzl-1 were removed and inoculated with and without *S. sclerotiorum*, and the seedlings treated with Tween-80 were also inoculated with and without *S. sclerotiorum*. Three replicates (20 leaves per replicate) were included for each treatment. Leaf disinfection treatment and phytopathogen inoculation were performed according to the description provided in Section 2.6, in which the strain AnS1Gz1-1 was replaced by *S. sclerotiorum* (cultured for 5–7 days on PDA medium). The incidence rate of soybean leaves was observed and recorded at 4, 5, 6, and 7 days post-inoculation (dpi); the disease incidence was calculated as follows: incidence rate (%) = (number of incidents/total number of inoculated plants) × 100 (Li et al., 2023). The diameter of disease spots on diseased leaves in each treatment was calculated by the cross method at 4 dpi (Sui et al., 2022).

### 2.10 Determining the effect of fungal colonization in soybean on insect feeding selectivity

A sterile plastic culture dish with a diameter of 14 cm was lined with filter paper, an appropriate amount of distilled water was added to keep the filter paper moist, and five pieces of 1 cm × 1 cm soybean leaves colonized or not colonized with fungal strain AnS1Gz1-1, of the same size along the circumference, were spaced evenly on the plate. For the non-selective test, five pieces of colonized and not colonized leaves were placed in different Petri dishes. In both selective and non-selective experiments, 20 s instar larvae of *S. litura* were inoculated in the center of the filter paper and transferred to a feeding room at 26 ± 1°C with a relative humidity of 60 ± 10% and a 14 h light:10 h dark photoperiod. Both experiments were repeated five times. The number of larvae on each leaf was recorded at 1, 1.5, 2, 2.5, 3, 3.5, 4, 4.5, 5, 5.5, and 6 h after inoculation, and the feeding rate of pests on soybean leaves was calculated using the following formula (Russo et al., 2019):

Feeding rate (%) = (number of insects feeding on soybean leaves/20) × 100.

### 2.11 Statistical analysis

All experimental data were analyzed using IBM SPSS Statistics 25 software (IBM Corp., Armonk, United States) for one-way analysis of variance (ANOVA; *p* < 0.05) and plotted using GraphPad Prism 8.0.2 (GraphPad Software Inc., San Diego, United States).

## 3 Results

### 3.1 Morphological and molecular identification of the EPF strain

**Figure 1:**
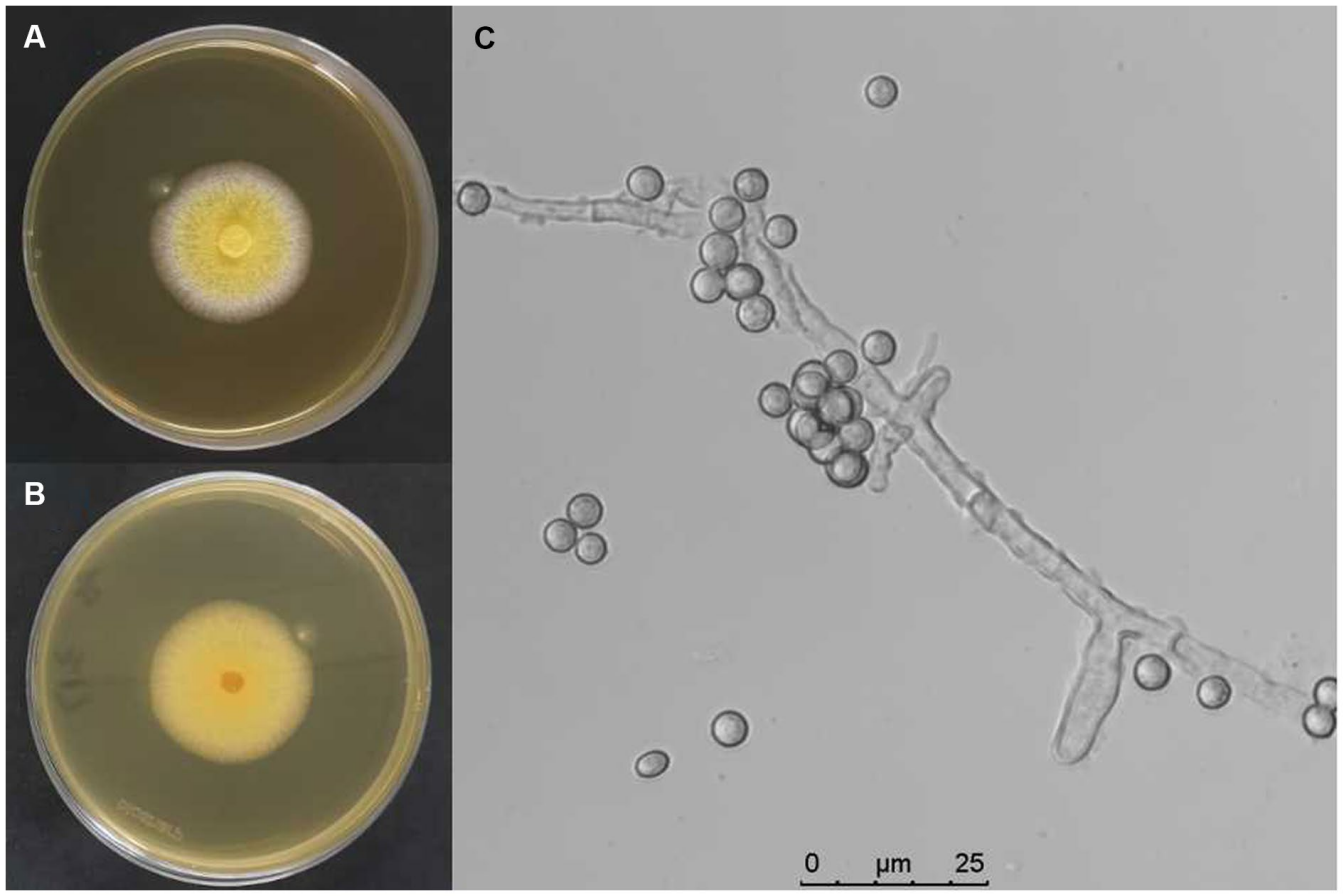
Biological characteristics of *A. nomiae* strain AnS1Gzl-1. **(A)** Colony front on PDA medium after inoculation for 10 days. **(B)** Colony back on PDA medium after inoculation for 10 days. **(C)** Conidia and hyphae of the fungal strain.

The infected fungal strain from muscardine fungus-infected larvae of *S. litura* in the soybean field was isolated and purified, designated as AnS1Gz1-1. The fungal strain was cultured on PDA medium with antibiotic, and colonies were yellow (Figures 1A,B). Observation under a light microscope revealed that conidia were single-celled and rough-walled with a diameter of spheres measuring approximately 3–5 μm in size, and hyphae were colorless, separated, and branched (Figure 1C). This morphology matched the description of the fungus given by Zhou et al. (2020).

**Figure 2:**
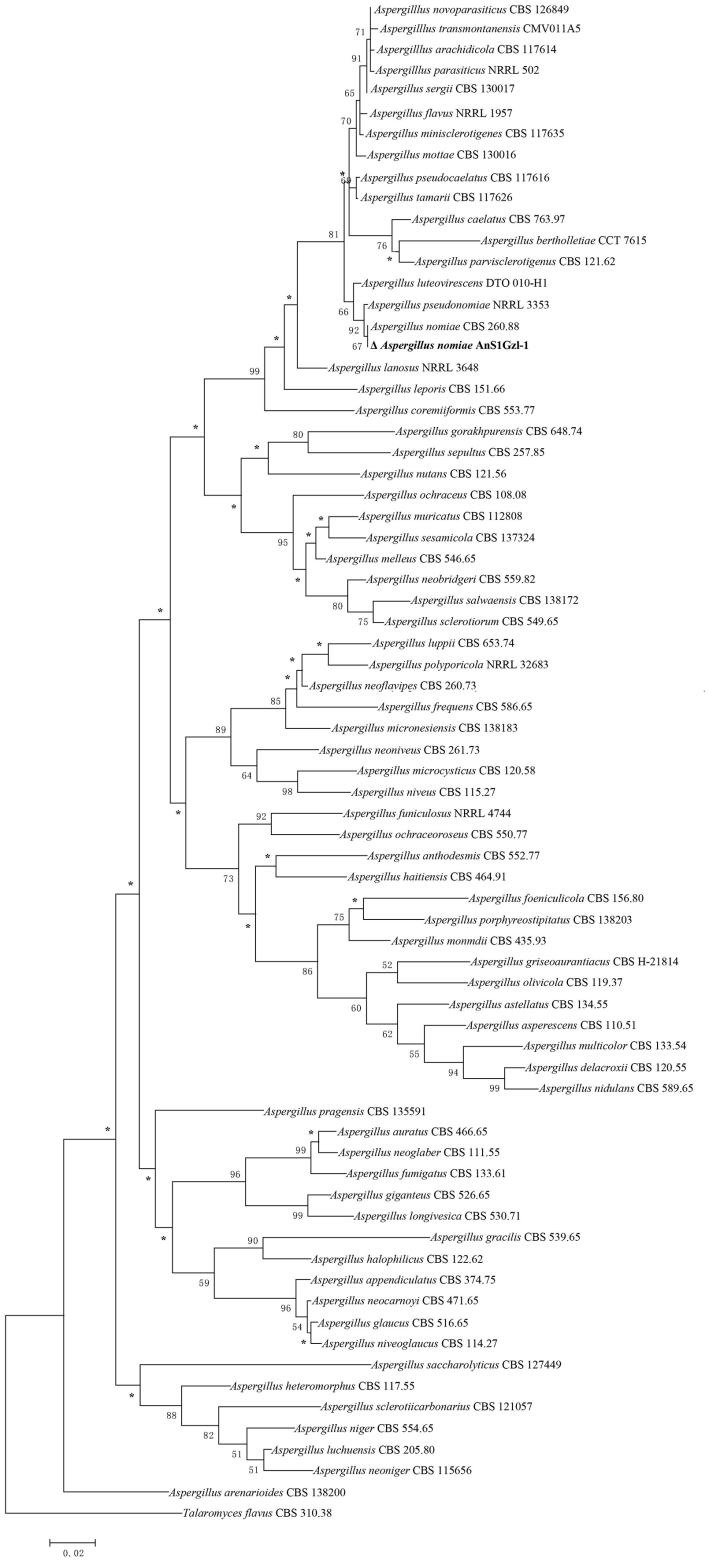
Phylogenetic analysis of combined *ITS*, *BenA,* and *CaM* sequence data. Numbers at branches represent bootstrap values from 1,000 replicates in the maximum likelihood method analysis, respectively. The scale represents genetic distance. *Branches support below 50%.

DNA fragment sequencing results showed that the length of genes ITS, BenA, and CaM of the strain were 586, 433, and 528 bp, respectively. The DNA sequences were submitted to GenBank and assigned the accession numbers OR425151.1, OR450636.1, and OR450635.1, respectively. The DNA sequences by Blastn comparison in Genbank showed that the strain was 99–100% similar to the reported *A. nomiae* strain. Phylogenetic tree analysis indicated that the strain clustered with the *A. nomiae* strain CBS 260.88 (Figure 2), which confirmed our morphological identification that the strain belongs to *A. nomiae*.

### 3.2 Pathogenicity of strain AnS1Gz1-1 against insect pests

**Figure 3:**
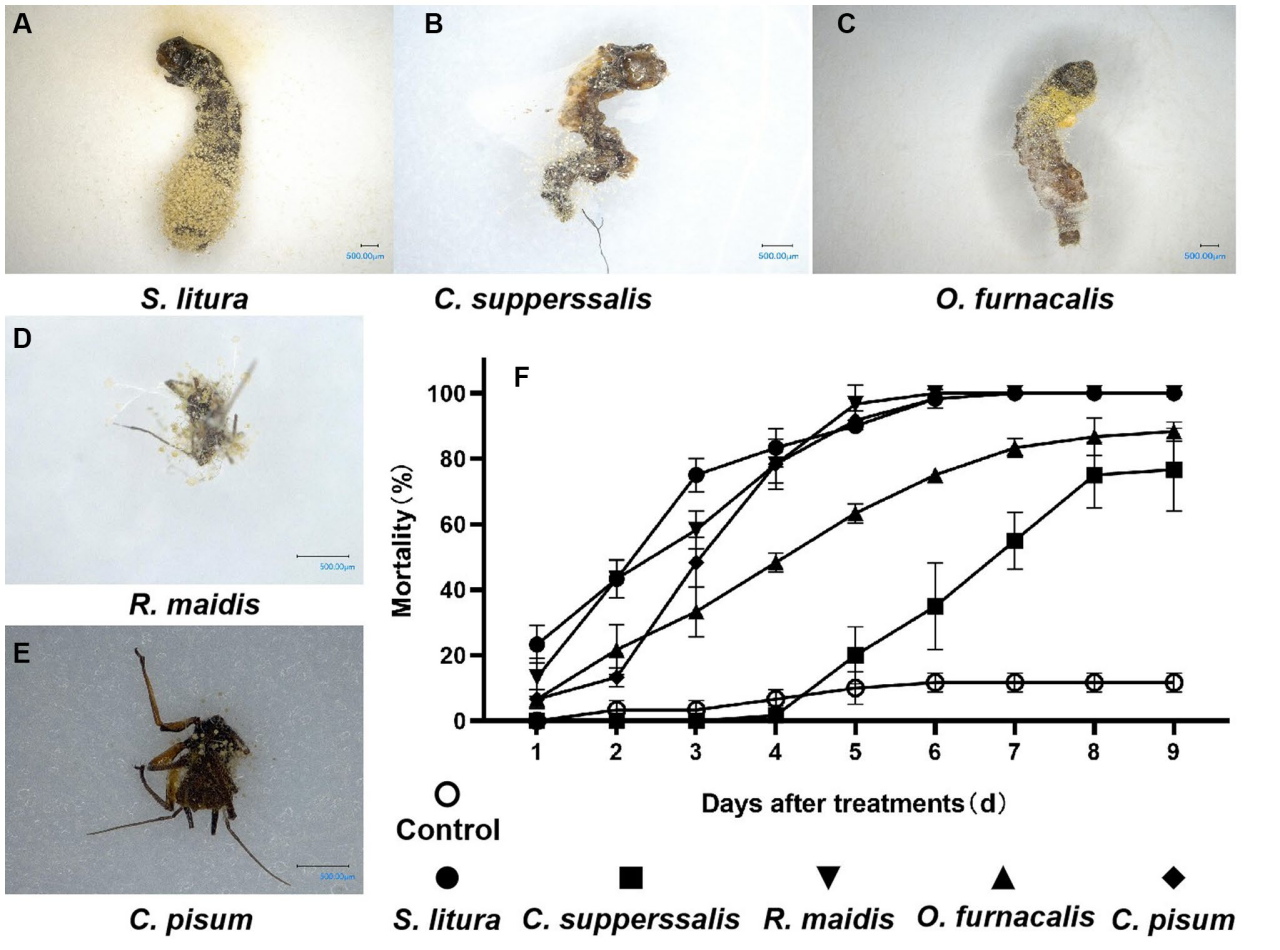
Virulence evaluation of *Aspergillosis nomiae* strain AnS1Gzl-1 against insect pests. **(A–E)** Muscardine cadavers of five kinds of insect pests infected by *A. nomiae* strain AnS1Gzl-1. **(F)** Corrected mortality of tested insects following infection by *A. nomiae* strain AnS1Gzl-1. Different lowercase letters indicate significant differences in corrected death rates of different strains tested by Duncan’s new complex range method (*p* < 0.05).

The pathogenicity of strain AnS1Gz1-1 against five important agricultural insect pests was explored. The results showed that AnS1Gz1-1 infection caused muscardine cadavers for all tested insects, whose body surface was covered with clearly visible conidia (Figures 3A–E), the colony and conidia morphologies following reisolation from dead insect bodies were consistent with those of strain AnS1Gz1-1. The EPF strain AnS1Gz1-1 showed high but varying virulence against the five tested insects. The pathogenicity of strain AnS1Gz1-1 toward *R. maidis*, *S. litura*, and *C. pisum* was strongest; the mortality rate of *S. litura* treated with the fungus was 90% at 5 days post-inoculation, the mortality rate of *O. furnacalis* treated with the fungus was 80% at 8 days post-inoculation, and the weakest virulence was to *C. supperssalis*, which mortality rate less than 80% at 9 days post-inoculation (Figure 3F). LT50 was determined to establish the pathogenicity of strain AnS1Gzl-1 against all five insect pests (Table 1), and the lowest LT50 of 2.212 days was against *S. litura*, while the highest was 5.376 days against *C. supperssalis*, and virulence against the two Hemiptera insect pests (*R. maidis* and *C. pisum*) was relatively high with LT50 values of 2.524 and 3.128 days, respectively.

**Table 1:** Virulence of *Aspergillosis nomiae* strain AnS1Gz1-1 toward various insect pests.

Tested insects	Spores/mL	LT50 (d)	95% confidence intervals (days)	c^2^Chi-square test c^2^	Slope ± SE
*S. litura*	1 × 10^8^	2.212	1.867–2.505	8.373	0.552 ± 0.052
*C. supperssalis*	1 × 10^8^	5.376	4.892–5.864	97.832	0.422 ± 0.024
*O. furnacalis*	1 × 10^8^	4.374	3.992–4.737	12.280	0.332 ± 0.026
*R. maidis*	1 × 10^8^	2.542	2.278–2.784	10.319	0.675 ± 0.062
*C. pisum*	1 × 10^8^	3.128	2.905–3.345	8.790	0.783 ± 0.065

### 3.3 Antagonistic activity of AnS1Gzl-1 against phytopathogens *in vitro*

**Figure 4:**
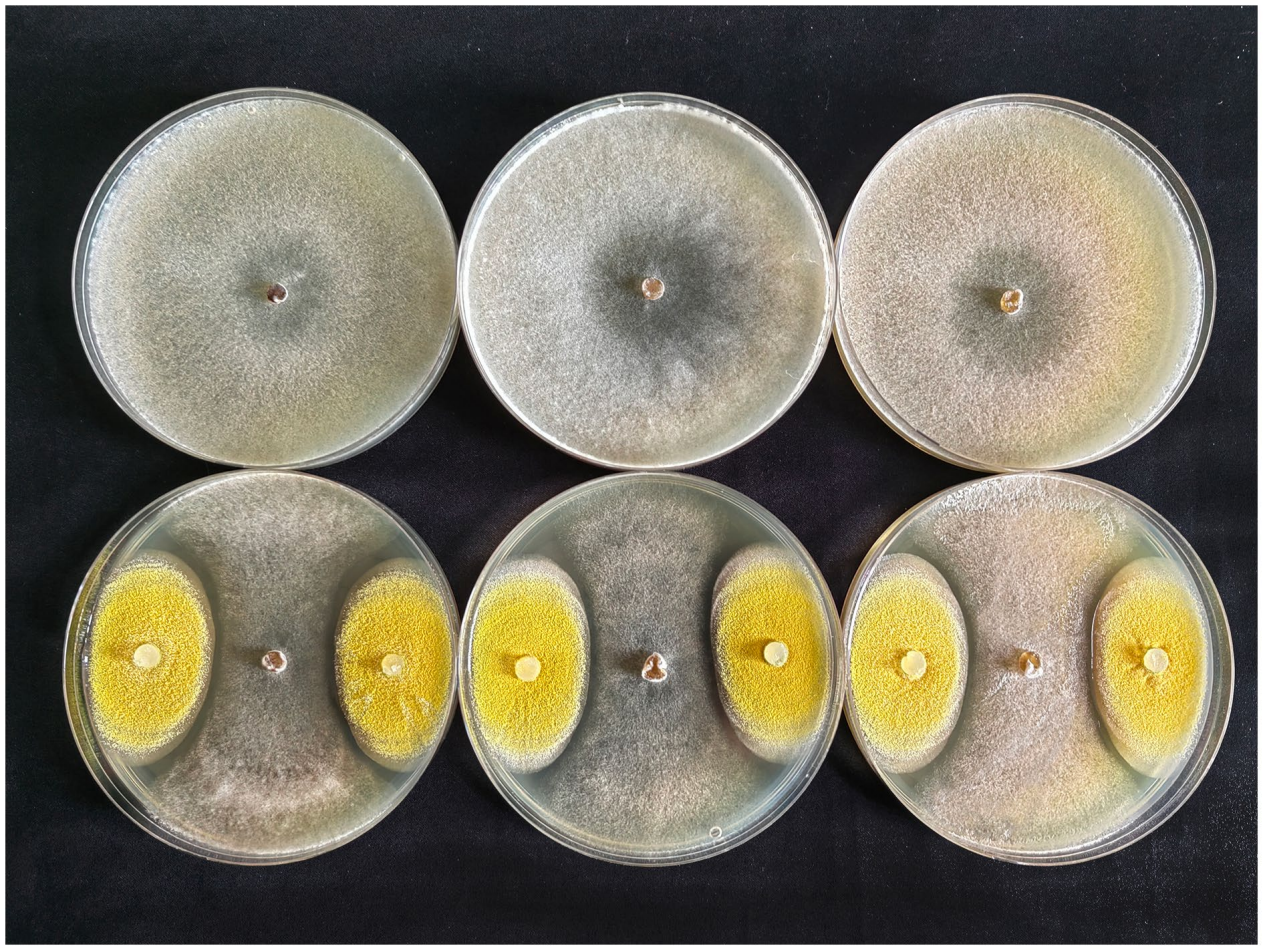
Confrontation cultures of *Sclerotinia sclerotiorum* and *Aspergillosis nomiae in vitro* at 5 days post-inoculation. The top row corresponds to *S. sclerotiorum* colonies cultured on medium, and the bottom row corresponds to confrontation cultures of *S. sclerotiorum* and *A. nomiae* on PDA medium.

After inoculation on PDA medium, colonies of *S. sclerotiorum* and EPF strain gradually expanded over time, and when they met, an obvious antagonistic circle was observed around EPF colonies, while colony growth of *S. sclerotiorum* cocultured with EPF strain was lower than when cultured alone (Figure 4). This indicates that *A. nomiae* could significantly inhibit the growth of *S. sclerotiorum in vitro*.

### 3.4 Colonization of EPF strain in soybean plants

**Figure 5:**
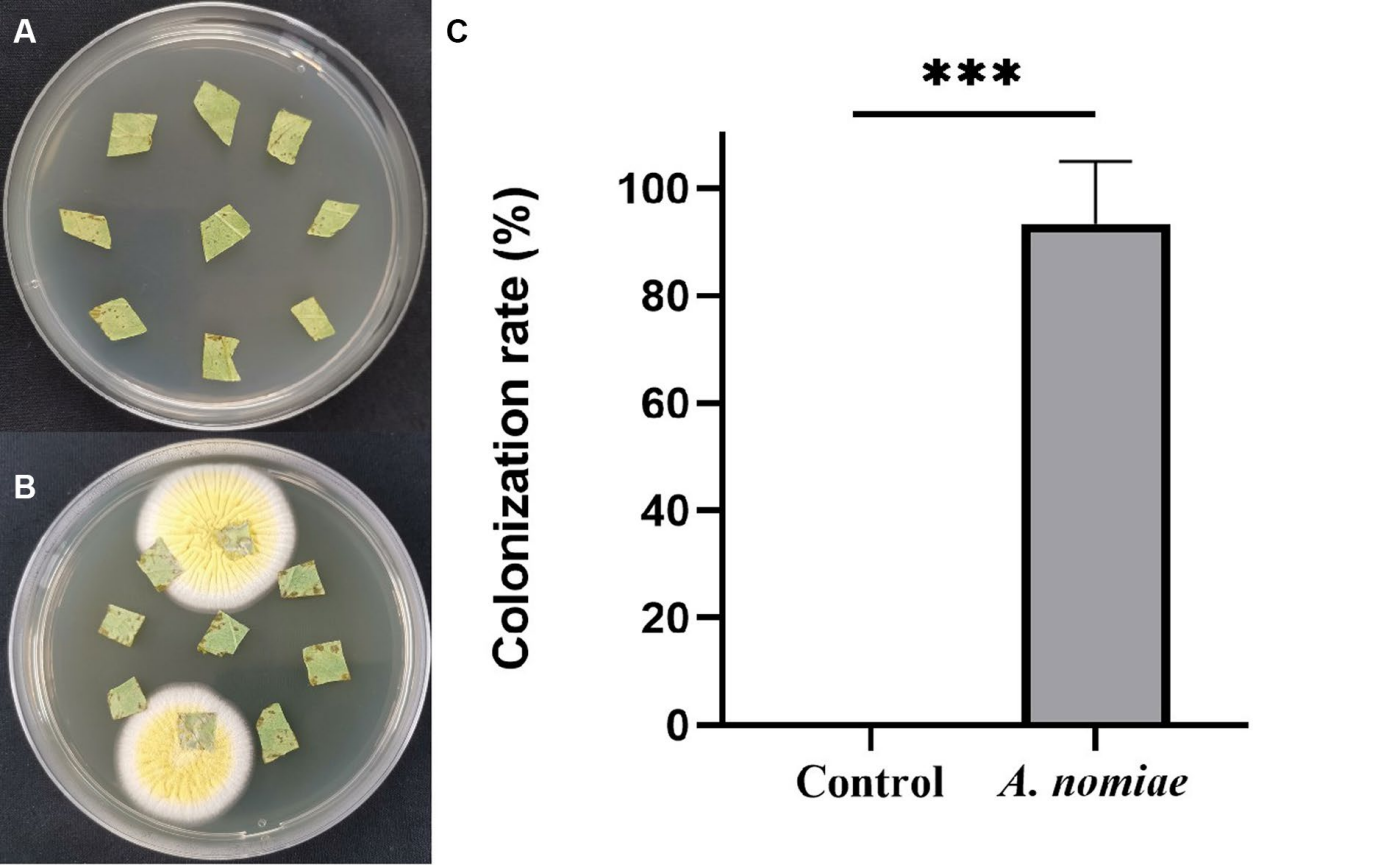
Colonization detection of *Aspergillosis nomiae* strain AnS1Gzl-1 in soybean plants. **(A)** Blank controls. **(B)** Inoculated with *A. nomiae* strain AnS1Gzl-1. **(C)** Colonization rate (ANOVA, ***p < 0.001).

After root irrigation with strain AnS1Gzl-1, leaf pieces of inoculated and non-inoculated plants with EPF were cultured on a PDA medium. On day 4 of cultivation, white fungal mycelium began to grow from the edge of soybean leaf tissue irrigated with the AnS1Gzl-1 strain, which was not observed in controls (Figures 5A,B), indicating that the AnS1Gzl-1 strain had the ability to colonize soybean plants through root irrigation. As shown in Figure 5C, the colonization rate of the fungus in soybean plants was 90%.

### 3.5 Endophytic colonization of *Aspergillus nomiae* affects insect pest feeding

**Figure 6:**
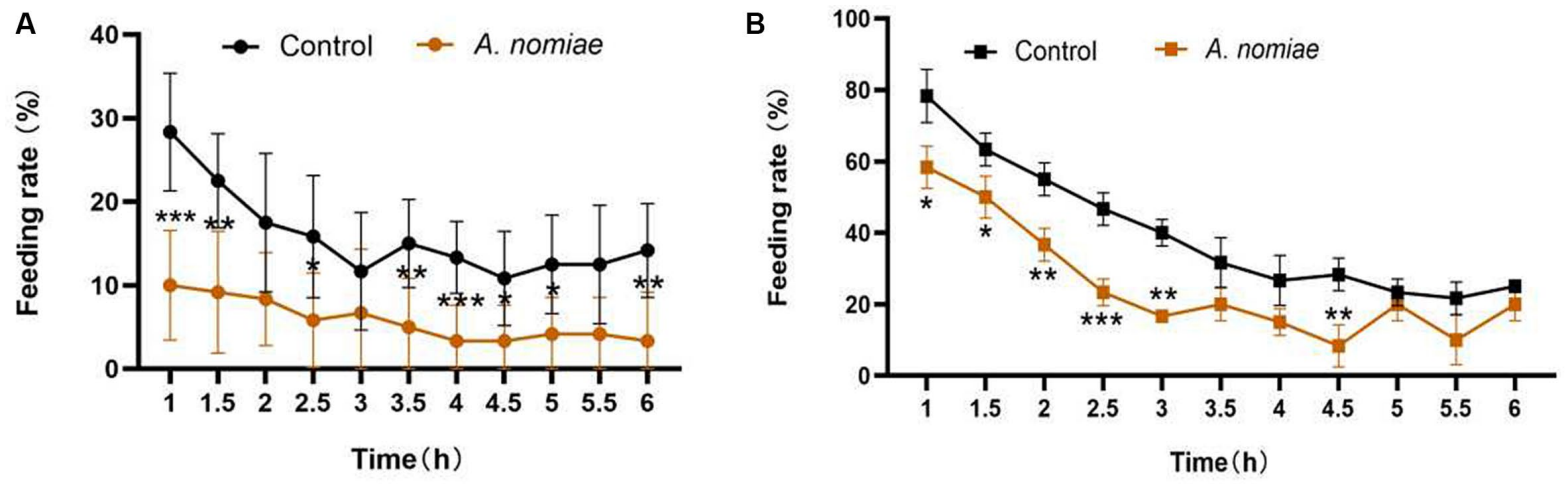
Feeding selectivity of second instar larvae of *Spodoptera litura* on *Aspergillosis*-soybean symbiosis. **(A)** Feeding rate of leaves with and without *A. nomiae* colonization in the selective test. **(B)** Feeding rate of leaves with and without *A. nomiae* colonization in the non-selective test (ANOVA, *p < 0.05, **p < 0.01, ***p < 0.001).

In the feeding experiment, within 6 h after inoculation, the feeding rate of second instar larvae of *S. litura* on leaves of the control group was consistently higher than that on leaves of the *A. nomiae* colonization group in either selective (Figure 6A) or non-selective (Figure 6B) experiments. Endophytic colonization of *A. nomiae* had a deterrent effect on *S. litura* larvae feeding.

### 3.6 Colonization by AnS1Gzl-1 induces plant resistance against *Sclerotinia sclerotiorum*

**Figure 7:**
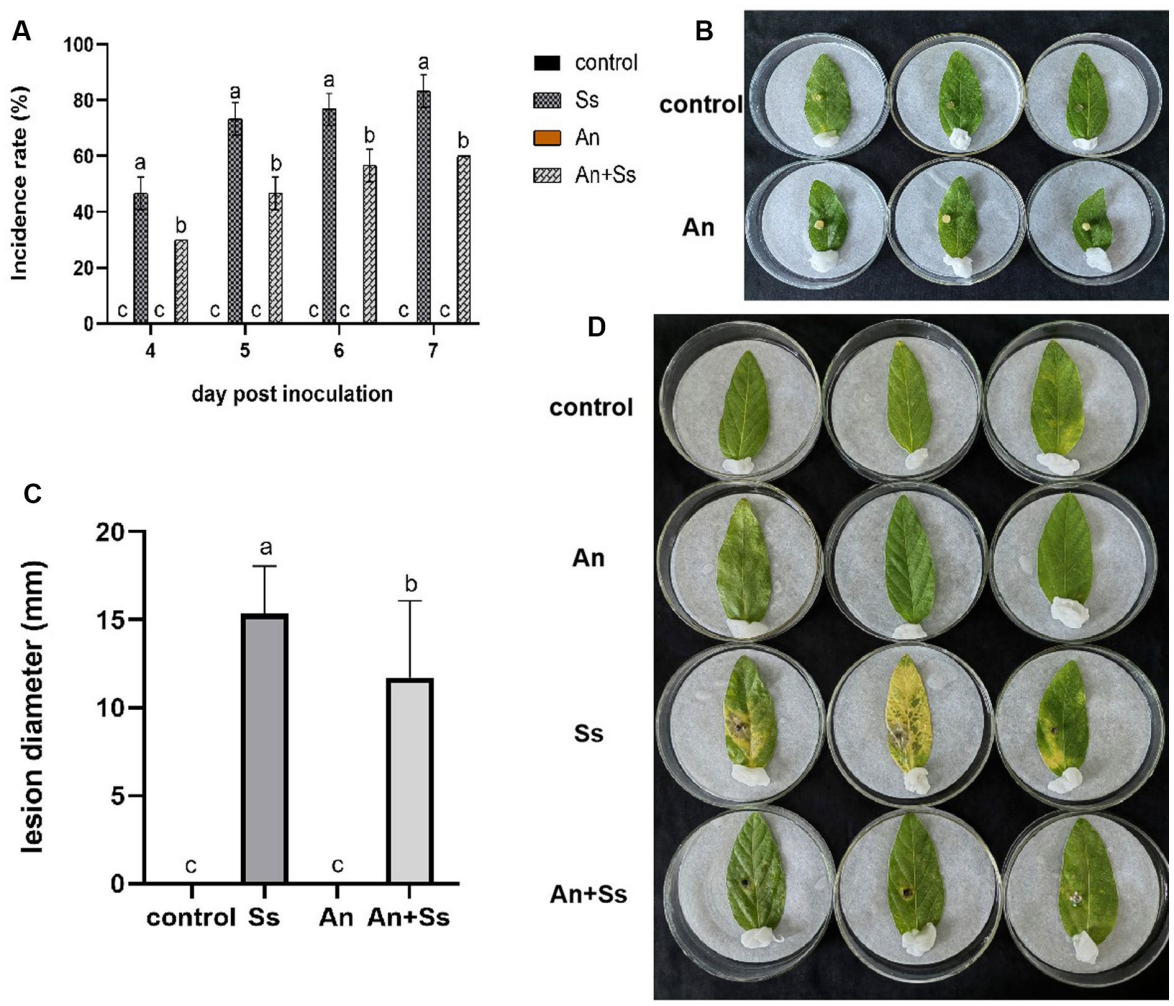
Disease resistance induction by *Aspergillosis nomiae* as an endophyte. **(A)** Incidence rate of different treatments at different times following phytopathogen inoculation. **(B)** Symptoms of *A. nomiae* inoculated soybean leaves. **(C)** Lesion diameter of different treatments on day 4 following phytopathogen inoculation. **(D)** Symptoms of soybean leaves for different treatments at 4 days after phytopathogen inoculation. Different lowercase letters indicate significant differences in corrected death rates of different strains tested by Duncan’s new complex range method (*p* < 0.05).

*In vitro* assays were performed to determine the resistance of *A. nomiae* against *S. sclerotiorum* following endophytic colonization in plants. The results showed that on 3 days post-inoculation of *S. sclerotiorum*, soybean leaves began to suffer from disease. Over time, the disease incidence rate on leaves was increased in both the *A. nomiae* colonization group and the control group, but compared with controls, the incidence rate of leaves colonized by *A. nomiae* was significantly lower. After 7 days post-inoculation, the incidence rate on soybean leaves in the group inoculated with *S. sclerotiorum* alone was 83.33 ± 5.77%, compared with 60.00 ± 0.00%(*p <* 0.001, *F* = 27.500) in the *A. nomiae* colonization group (Figure 7A).

On day 3 post-inoculation, soybean leaves showed no symptoms of pathogen infection in either strain AnS1Gzl-1 (An) or culture groups, which means *A. nomiae* had no pathogenicity to soybean leaves (Figure 7B). For the assay of induced disease resistance by AnS1Gzl-1 colonization, on day 4 post-inoculation, the diameter of the disease lesions on soybean leaves in the *A. nomiae* colonization group (An+Ss) was significantly smaller than that in the group inoculated only with the pathogen (Ss). The control and *A. nomiae* colonization (An) groups exhibited no symptoms, and hence, *A. nomiae* colonization was not harmful to plants (Figures 7C,D). Symptoms of soybean leaves displayed only partial yellowing around pathogen inoculation sites in the *A. nomiae* colonized groups (An+Ss), whose lesion diameter on day 4 following phytopathogen inoculation was 11.72 ± 4.36 mm, significantly smaller than that of the phytopathogen inoculation only group (Ss), which was 15.36 ± 2.70 mm (*p <* 0.001, *F* = 6.918), while almost the half to whole of leaves displayed yellow color in the group inoculated with pathogen alone (Figure 7D).

## 4 Discussion

EPF strains are important biological control agents for insect pests that cause significant yield loss and quality decline in agriculture and forestry. In previous studies, research on EPF resources mainly focuses on *Beauveria* spp., *Metarhizium* spp., and *Lecanicillium* spp. (Bamisile et al., 2021). Abundant EPF resources can provide more efficient biological control of pests. It is necessary to constantly explore new EPFs with high efficiency and broad spectrum. *Aspergillus* species are diverse fungi that are widely distributed in nature with multiple biological effects. The biocontrol effect on insect pests of some *Aspergillus* species has been reported, such as *A. flavus*, *A. nomius*, *A. fijiensis,* and *A. oryzae*, which could be used to control *Locusta migratoria*, *S. litura*, *Diaphorina citri*, and *Dolichoderus thoracicus* (Zhang et al., 2015; Karthi et al., 2018; Lin et al., 2021; Yan et al., 2022). It has been reported that *A. nomiae* is not only morphologically similar to *A. flavus*, but also can cause fatal rhinofacial infection in humans, but with resistance to antifungal amphotericin B (AMB) (Zhou et al., 2020). Here, as the first record, we isolated and identified a strain of *A. nomiae* AnS1Gzl-1 that caused muscardine cadaver of *S. litura* larvae naturally, with relatively high pathogenicity to a variety of agricultural insect pests, and can be used as a new broad-spectrum EPF for insect pest control. Importantly, further research is needed to determine whether the pathogenic mechanism of *A. nomiae* on insects is the same as that of humans, especially, whether its application as a biological insecticide in the field will pose a risk of infection to humans and animals. Nevertheless, our molecular identification results indicated that *A. nomiae* AnS1Gzl-1 is more closely related to *A. pseudonomiae* (a non-pathogenic pathogen) in evolution, while it is more distant from *A. flavus*. The results are similar to a previous study from Hatmaker et al. (2022). There were only 46 secondary metabolite gene synthesis clusters related to the infection in its genome and it was 16–20 fewer compared with that in the pathogenic *A. flavus*.

Previous studies have proven that EPF can not only kill insect pests, but also establish obvious biological effects against insect pests via colonization in plants, including toxicity, developmental disorders, and avoidance. For example, endophytic colonization by *B. bassiana* and *M. anisopliae* in maize plants affects the fitness of *S. frugiperda*, and both strains negatively impact the development and fecundity of *S. frugiperda* (Altaf et al., 2023). Sinno et al. (2021) found that aphids reared on *B. bassiana*-treated plants for their entire lifespan were negatively affected in terms of survival and fertility in comparison with control cohorts; Mseddi et al. (2022) screened a wild strain of *B. bassiana* B12 that not only promoted several plant growth parameters after root inoculation, but also displayed toxicity toward *A. gossypii* following colonization in cotton plants. Zhu et al. (2023) found that *B. bassiana*-inoculated maize plants can influence the eggs laying selectivity of *O. furnacalis* females by changing plant volatile profiles; and González-Mas et al. (2021) found that when the cotton was colonized by *B. bassiana*, they emit a different blend of volatile compounds compared to uncolonized control plants, which can affect the feeding behavior of insect pests *A. gossypii*, *S. frugiperda*, and *S. littoralis*. Here, we have similar results that *A. nomiae* had the ability of plant colonization and showed a significant repellent effect on *S. litura* larvae, an important insect pest on soybean, in both selective and non-selective assays. In short, it indicates that *A. nomiae* not only directly kills insects but also has the potential to achieve ecological control of pests through endogenous colonization.

*A. nomiae* was also known as a causal agent of plant disease due to its high incidence in Brazil nuts (*Bertholletia excelsa* H.B.K.) and its strong production of carcinogenic metabolites (Reis et al., 2022). In the present study, we found that *A. nomiae* strain AnS1Gzl-1 causes no damage to plants by either inoculating it on the surface of soybean leaves or colonizing it in plant tissues through root irrigation. However, *A. nomiae* strains have been reported to produce both series B and G aflatoxins (Ehrlich et al., 2004), which are notorious for hepatocellular carcinoma development, lung adenocarcinoma, and chronic inflammatory changes (Liu et al., 2015); hence, it is necessary to clarify whether toxins are metabolized by *A. nomiae* strain AnS1Gzl-1 in its endophytic plants in a future study.

Numerous EPF strains have a plant disease control effect via endogenous colonization (Bamisile et al., 2018; Vega, 2018). Some strains showed no directly antagonistic effects against pathogens *in vitro* but can improve plant resistance to diseases following endogenous colonization by altering the phyllosphere microbiome of maize after colonization (Chang et al., 2021) or regulating salicylic acid (SA) and jasmonic acid (JA) pathways (Qin et al., 2021; Gupta et al., 2022). Nevertheless, EPF exhibited direct antagonism against pathogens via antibiosis, competition, or parasitism through the production of fungal secondary metabolites (Ownley et al., 2008; Vega et al., 2009). Previous research has found that *Aspergillus* species could produce various beneficial compounds such as cyclosporine A, asperfuranone, terrrein, itaconic acid, kojic acid, citric acid, and gluconic acid (Sun et al., 2018; Wu et al., 2019; Chigozie et al., 2022), which showed antifungal, antibacterial, and phytotoxic activities, and played a crucial role to antagonize the devastating phytopathogens (Ding et al., 2019). It was found that *A. versicolor* had the ability to inhibit the destructive phytopathogen *Macrophomina phaseolina*. In dual culture bioassays, *A. versicolor* showed potential antagonist activity and reduced the pathogen’s growth by 60% over control, with the mechanism of which secondary metabolites can degrade the DNA of pathogenic fungi (Khan and Javaid, 2022). Herein, *A. nomiae* strain AnS1Gzl-1 inhibited *S. sclerotiorum* growth on the PDA medium as evidenced by an obvious antagonistic zone, and it significantly improved resistance of plants against *S. sclerotiorum* infection on soybean leaves following colonization. Furthermore, it is necessary to clarify whether there are antagonistic products metabolized from *A. nomiae* that inhibit the growth of phytopathogens in future studies. To the best of our knowledge, there have been few reports on the biocontrol effects against soil-borne diseases and their causal agents induced by endophytic colonization of EPF; only *Metarhizium* spp. and *B. bassiana* have been mentioned for the control of *Fusarium culmorum*, *Rhizoctonia* spp., and *S. sclerotiorum* in the literature (Jaber, 2018; Raad et al., 2019; Tomilova et al., 2020; Deb et al., 2023). Here, we found that *A. nomiae* strain AnS1Gzl-1 has great potential for sclerotinia disease control, although its mechanism of resistance requires further investigation.

In summary, we isolated a strain of *A. nomiae* from diseased *S. litura* larvae on soybean in a field, which had broad-spectrum direct insecticidal effects on various insect pests. It is the first record that *A. nomiae* is an EPF and has potential as a biological insecticide. Another important finding, the strain could indirectly control insect pests and phytopathogen damage via endophytic colonization without damage to host plants like other EPFs.

## Data availability statement

The datasets presented in this study can be found in online repositories. The names of the repository/repositories and accession number(s) can be found in the article/supplementary material.

## Author contributions

ZZ: Conceptualization, Funding acquisition, Writing – original draft. YT: Investigation, Methodology, Validation, Writing – original draft. LS: Formal analysis, Resources, Software, Writing – original draft. YL: Data curation, Methodology, Validation, Writing – original draft. KC: Data curation, Software, Visualization, Writing – original draft. YZ: Data curation, Investigation, Writing – original draft. QL: Conceptualization, Writing – review & editing. WS: Conceptualization, Visualization, Writing – review & editing.
